# Monitoring Initiation and Administration of Covert Medication(s) for Service Users in Amber Ward of Millbrook Mental Health Unit, Nottinghamshire Healthcare NHS Foundation Trust: A Re-Audit of Compliance With Maudsley Prescribing Guidelines in Psychiatry

**DOI:** 10.1192/bjo.2024.573

**Published:** 2024-08-01

**Authors:** Silvester Hammed, Adam Kinch, Anto Rajamani

**Affiliations:** Nottinghamshire Healthcare NHS Foundation Trust, Nottingham, United Kingdom

## Abstract

**Aims:**

1. To evaluate standards of practice regarding initiation and administration of covert medication(s), with comparison to the previous audit completed in January 2021.
2. To highlight improvements and weaknesses requiring further recommendations for effective future practice.

**Methods:**

This clinical audit assessed the current practice in Amber Ward (Old Age Ward for Dementia patients) against the same standards of practise used in the previous audit.

The Audit Checklist included 10 standards from Maudsley prescribing guidelines for Covert Medication Pathway.

A retrospective review of the paper and electronic records of 21 service users initiated on a covert medication plan between January 2021 and June 2022 was carried out.

A descriptive statistic on the data and presented results in tables comparing frequencies and percentages with the data from previous audit was then performed.

**Results:**

1. An increase in percentage of documented evidence of covert medication plan being discussed with a relative with Lasting Power of Attorney or in a Best Interest Meeting to 95% (n = 20) from 85% (n = 12) in the previous audit.
2. An increase in percentage of documented evidence of pharmacy input on covert medication administration plan to 100% (n = 21) from 47% (n = 7) in the previous audit.
3. An increase in percentage of documented evidence of covert medication administration in the drug charts to 100% (n = 21) from 53% (n = 8) in the previous audit.
4. An increase in percentage of documented evidence of covert medication review date on the covert medication initiation forms to 85% (n = 18) from 67% (n = 10) in previous audit.
5. A decrease in percentage of documented evidence of MDT discussion prior to starting covert medication plan to 90% (n = 19) from 100% (n = 15) in previous audit.

**Conclusion:**

This re-audit showed some improvement with 100% compliance in 4 out of 10 standards, however, there's still room for improvement to get the compliance to 100% across all the standards.

We therefore recommended strict adherence to existing covert medication initiation plan form, with particular attention to be paid to the standard of proper documentation of the details of MDT discussions around covert medication plan initiation, as there was surprisingly a reduction noted in this standard.

Finally, we recommended that another re-audit be considered within 2 years of completion of this re-audit.

